# Role of the Organic Cation in 2D Chiral Hybrid Palladium Chloride Materials

**DOI:** 10.1021/acs.inorgchem.5c04105

**Published:** 2025-11-10

**Authors:** Zheng Zhang, Daniel B. Straus

**Affiliations:** Department of Chemistry, 5783 Tulane University, New Orleans, Louisiana 70118, United States

## Abstract

We report a series of new chiral and achiral organic–inorganic hybrid palladium chlorides and explore the influence of subtle changes to the organic cation on the structural, electronic, and chiroptical properties. These materials are (C_4_H_10_N)_2_PdCl_4_ ((C_4_H_10_N)^+^ = pyrrolidinium), R/S/racemic-(C_4_H_9_FN)_2_PdCl_4_ ((C_4_H_9_FN)^+^ = 3-fluoropyrrolidinium), and R/S/racemic-(C_5_H_12_N)_2_PdCl_4_ ((C_5_H_12_N)^+^ = 3-methylpyrrolidinium). R/S-(C_4_H_9_FN)_2_PdCl_4_ and R/S-(C_5_H_12_N)_2_PdCl_4_ are chiral metal halides, while (C_4_H_10_N)_2_PdCl_4_ and the racemic materials are achiral. Structurally, these materials are distorted Ruddlesden–Popper phase 2D perovskites. Pd^2+^ sits at the center of a distorted octahedron with four nearly identical 2.3 Å Pd–Cl bonds and two long (3.7–4.2 Å) Pd–Cl contacts. The long Pd–Cl contacts cause these materials to exhibit quasi-zero-dimensional electronic behavior with limited electronic coupling between Pd atoms. R- and S-(C_4_H_9_FN)_2_PdCl_4_ show appreciable circular dichroism signals, whereas R- and S-(C_5_H_12_N)_2_PdCl_4_ do not. We hypothesize that this difference originates from much stronger interactions between (C_4_H_9_FN)^+^ cations and the inorganic framework in R/S-(C_4_H_9_FN)_2_PdCl_4_ than in R/S-(C_5_H_12_N)_2_PdCl_4_, which is consistent with our observation that the R and S cations occupy distinct positions in racemic (C_4_H_9_FN)_2_PdCl_4_ but form a solid solution in racemic (C_5_H_12_N)_2_PdCl_4_. Our results highlight how small differences in the cation greatly affect the structural, electronic, and chiroptical properties of palladium­(II) chloride materials.

## Graphical abstract

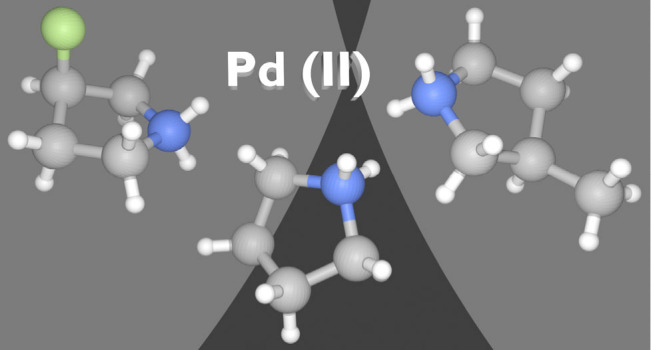

## 1 Introduction

Metal halide perovskites with the general chemical formula ABX_3_ (A = methylammonium (CH_3_NH_3_)^+^, formamidinium [HC­(NH_2_)_2_]^+^, or Cs^+^; B = Pb^2+^; X = I^–^, Br^–^, or Cl^–^) have emerged as promising materials for a number of practical applications, including radiation detection, photodetection, and light-emitting diodes.^1–3^ If an enantiomerically pure chiral organic cation is used,^4^ chirality is transferred to the inorganic metal halide framework,^5,6^ and chiral metal halide materials have demonstrated potential for spin-related optoelectronic and circularly polarized light detection applications.^7–10^ A variety of chiral metal halides, including lead-based organic–inorganic hybrid R/S-(MBA)_2_PbI_4_ (MBA = α-methylbenzylammonium), R/S-(2AEP)­Pb_2_I_6_ (2AEP = 2-(1-aminoethyl)­pyridin-1-ium), and R/S-(MBnP)­PbX_3_ (MBnP = methylbenzylpyridinium; X = I^–^, Br^–^, or Cl^–^), have been successfully prepared.^11–13^ The inclusion of lead in chiral halides, however, has posed great environmental concern.^14,15^ Therefore, lead-free halides with large chiroptical response are preferred, and reported lead-free chiral metal halides include R/S-(MBA)_2_SnI_4_, R/S-(1-PPA)_2_MnBr_4_ (1-PPA = 1-phenylpropan-1-amine), and R-(3-FPP)_2_SbCl_5_ (3-FPP = 3-fluoropiperidinium).^16–18^ Among the reported lead-free chiral halides, palladium­(II)-based chiral chlorides are rare. Examples of reported achiral palladium-based metal chlorides include two-dimensional (2D) layered (CH_3_NH_3_)_2_PdCl_4_,^19^ layered (C_8_H_17_NH_3_)_2_PdCl_4_,^20^ and (C_24_H_54_N_8_O_27_Si_16_)­Pd_2_Cl_5_.^21^ A few chiral hybrid palladium chloride materials also have been reported; based on our examination of the Cambridge Structural Database,^22^ R/S-(C_5_H_14_N_2_)­PdCl_4_ ((C_5_H_14_N_2_)^+^ = 2-methylpiperazinediium),^23^ R/S-(MBA)_2_PdCl_4_,^24^ and (C_4_H_10_N)­PdCl_3_ ((C_4_H_10_N)^+^ = pyrrolidinium)^25^ are the only reported chiral hybrid palladium chloride materials.

In this Article, we report a series of new chiral and achiral organic–inorganic hybrid palladium­(II) chloride materials with the general formula A_2_PdCl_4_ (where A is the organic cation) that allow us to understand how small changes to the organic cation affect the structure and chiroptical properties of the material. Specifically, we synthesized a new achiral material, (C_4_H_10_N)_2_PdCl_4_ ((C_4_H_10_N)^+^ = pyrrolidinium), and new chiral palladium­(II) metal chlorides based on (C_4_H_10_N)_2_PdCl_4_ where one of the 3-position hydrogens is replaced by F or CH_3_ (Figure 1): R/S-(C_4_H_9_FN)_2_PdCl_4_ ((C_4_H_9_FN)^+^ = 3-fluoropyrrolidinium), R/S-(C_5_H_12_N)_2_PdCl_4_ ((C_5_H_12_N)^+^ = 3-methylpyrrolidinium), and their racemic analogues. (C_4_H_10_N)_2_PdCl_4_ is a different phase of pyrrolidinium palladium chloride than the chiral variant with a formula of (C_4_H_10_N)­PdCl_3_ that we recently reported.^25^

**Figure 1:**
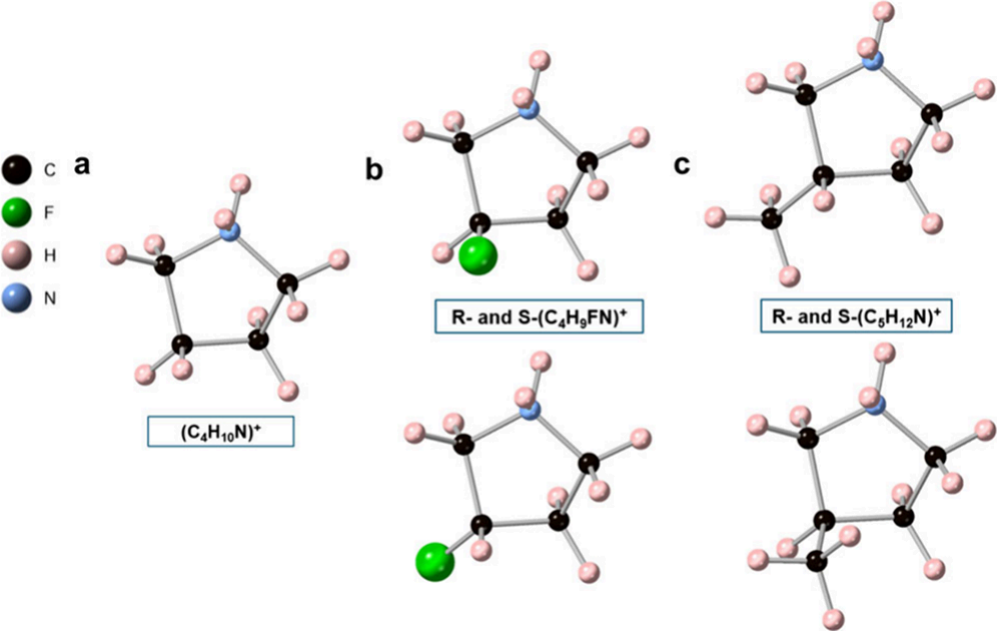
Structures of organic cations (a) (C_4_H_10_N)^+^, (b) R- and S-(C_4_H_9_FN)^+^, and (c) R- and S-(C_5_H_12_N)^+^.

Structurally, these materials can be depicted as distorted Ruddlesden–Popper phase 2D perovskites, where the inorganic PdCl_4_ layers are composed of corner-sharing distorted octahedra with the inorganic layers separated from one another by the organic cations. Within each PdCl_6_ octahedron, there are four short ∼2.3 Å Pd–Cl bonds with lengths independent of the cation and two long (3.7–4.2 Å) Pd–Cl contacts, the lengths of which are greatly affected by the choice of cation: as the organic cation becomes larger ((C_5_H_12_N)^+^ > (C_4_H_9_FN)^+^ > (C_4_H_10_N)^+^) (Figure 1), the two long Pd–Cl contacts also elongate. The materials’ electronic structure resembles that of quasi-zero-dimensional materials because the length of the long Pd–Cl contacts is comparable to the sum of the van der Waals radii of Pd^2+^ and Cl^–^,^26^ and this conclusion is supported by density functional theory (DFT) calculations. The two long Pd–Cl contacts are equivalent by symmetry in the achiral materials (C_4_H_10_N)_2_PdCl_4_, racemic (C_4_H_9_FN)_2_PdCl_4_, and racemic (C_5_H_12_N)_2_PdCl_4_. The nature of the substituted functional group affects how the cations behave in the racemic materials that contain a 1:1 ratio of R and S cations. In racemic (C_4_H_9_FN)_2_PdCl_4_, R and S cations occupy distinct positions within the crystal structure and can be specifically identified. In contrast, the cations in racemic (C_5_H_12_N)_2_PdCl_4_ form a solid solution, where there is a 50% chance that either R- or S-(C_5_H_12_N)^+^ occupies a given cation site. This difference emphasizes that interactions between the cations themselves and the inorganic framework are much stronger in racemic (C_4_H_9_FN)_2_PdCl_4_ than in racemic (C_5_H_12_N)_2_PdCl_4_ because of fundamental differences between F and CH_3_. Accordingly, Wallach’s rule, which states that crystals containing a racemic mixture are denser than those containing an enantiomerically pure sample,^27^ does not hold for (C_5_H_12_N)_2_PdCl_4_ because the racemic structure is less dense than its enantiomerically pure counterparts, which we attribute to less efficient packing within the crystal as a result of the cationic solid solution. The gap between the highest occupied molecular orbital (HOMO) and lowest unoccupied molecular orbital (LUMO) increases with the length of the long Pd–Cl contact. We also measure the chiroptical properties using circular dichroism (CD) spectroscopy and find that chiral R/S-(C_4_H_9_FN)_2_PdCl_4_ shows appreciable CD signals, potentially making this material suitable for chiroptical applications. As a comparison, R/S-(C_5_H_12_N)_2_PdCl_4_ does not. Our results highlight the role of the organic cation in determining the structural, electronic, and chiroptical properties in organic–inorganic 2D hybrid palladium­(II) chloride perovskites.

## 2 Results and Discussion

Crystals were obtained through the slow evaporation of hydrochloric acid from a solution containing PdCl_2_ and the organic cation (see the Experimental Methods). All grown crystals are plate-like and present a dark-orange or dark-brown color (Figure S1). The crystal structure of achiral (C_4_H_10_N)_2_PdCl_4_ is shown in Figure 2 and Figure S2. (C_4_H_10_N)_2_PdCl_4_ crystallizes in the orthorhombic *Pbca* (#61) space group (Table 1) and is a distorted Ruddlesden–Popper phase 2D perovskite,^28^ where there is a 1/2 unit cell shift in the position of Pd^2+^ in adjacent layers, similar to the materials (BA)_2_PbI_4_ (BA = butylammonium), (PEA)_2_PbI_4_ (PEA = phenethylammonium), and Cs_2_PbI_2_Cl_2_.^29–31^ The four short Pd–Cl bond distances in the inorganic PdCl_4_ layer are 2.31 Å in length (Figure 2b), consistent with the Pd–Cl bond distance values in other Pd-based metal halides such as (C_9_H_16_N_2_)­PdCl_4_ (2.313 Å), (C_5_H_14_N_2_)­PdCl_4_ (2.303 Å), and (C_8_H_12_N)_2_PdCl_4_ (2.295 Å),^23,24,32^ and slightly shorter than the sum of Shannon ionic radii for square planar Pd^2+^ and Cl^–^ ions, which is 2.45 Å.^33^ The long Pd–Cl bond contact within the PdCl_6_ pseudo-octahedral coordination is 3.7190(8) Å.

**Figure 2:**
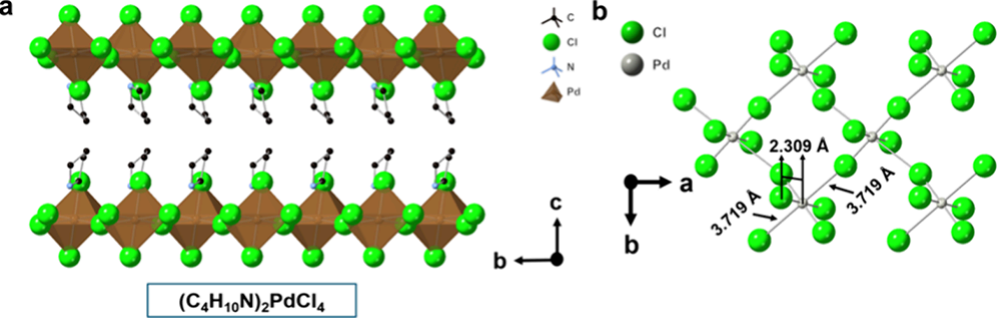
(a) Crystal structure of achiral metal halide (C_4_H_10_N)_2_PdCl_4_. (b) Structural view showing the Pd–Cl bond distance within the PdCl_4_ inorganic layer. H atoms have been omitted for the sake of clarity.

**Table 1:** Single-Crystal Data and Structural Refinement Parameters for Achiral (C_4_H_10_N)_2_PdCl_4_

formula weight (g/mol)	392.46
temperature (K)	300
wavelength (Å)	0.71073
crystal system	orthorhombic
space group	*Pbca* (#61)
*Z*	4
unit cell parameters	*a* = 9.2485(2) Å
	*b* = 7.5529(2) Å
	*c* = 21.5100(5) Å
	β = 90°
volume (Å^3^)	1432.68(6)
volume (Å^3^)/*Z*	358.17(1)
density (g/cm^3^)	1.820
absorption coefficient (μ) (mm^–1^)	2.015
θ_min_ – θ_max_ (deg)	5.932–72.724
no. of reflections collected	120 482
no. of independent reflections	3490
*R* ^*a*^ indices (*I* > 2σ(*I*))	*R*_1_ = 0.0416
	*wR*_2_ = 0.0948
goodness of fit on *F* ^2^	1.057
largest difference peak/hole (e^–^/Å^3^)	0.71/–1.16

Crystal structures of R/S/racemic-(C_4_H_9_FN)_2_PdCl_4_ are shown in Figure 3 and Figures S3–S5. R/S-(C_4_H_9_FN)_2_PdCl_4_ crystallizes in the monoclinic *P*2_1_ (#4) space group, and racemic (C_4_H_9_FN)_2_PdCl_4_ crystallizes in the monoclinic *P*2_1_/*c* (#14) space group (Table 2). Like (C_4_H_10_N)_2_PdCl_4_, R/S/racemic-(C_4_H_9_FN)_2_PdCl_4_ are distorted Ruddlesden–Popper phase 2D perovskites with similar connectivity; within a single PdCl_6_ pseudo-octahedron, each short Pd–Cl bond distance is 2.30–2.32 Å for R/S-(C_4_H_9_FN)_2_PdCl_4_ and racemic (C_4_H_9_FN)_2_PdCl_4_ (Figure 3). In R/S-(C_4_H_9_FN)_2_PdCl_4_, one of the two long Pd–Cl bond contacts is 3.70 Å in length, and the other is 3.80 Å. For racemic (C_4_H_9_FN)_2_PdCl_4_, the two long Pd–Cl bond contacts are both 3.70 Å in length and are equivalent by symmetry (Figure 3d). Furthermore, according to Wallach’s rule,^27^ racemic crystals tend to be denser than their enantiomerically pure counterparts, and this holds true for (C_4_H_9_FN)_2_PdCl_4_ (Table 2). The densities of R-(C_4_H_9_FN)_2_PdCl_4_, S-(C_4_H_9_FN)_2_PdCl_4_, and racemic (C_4_H_9_FN)_2_PdCl_4_ are 1.942, 1.938, and 1.969 g/cm^3^, respectively.

**Figure 3:**
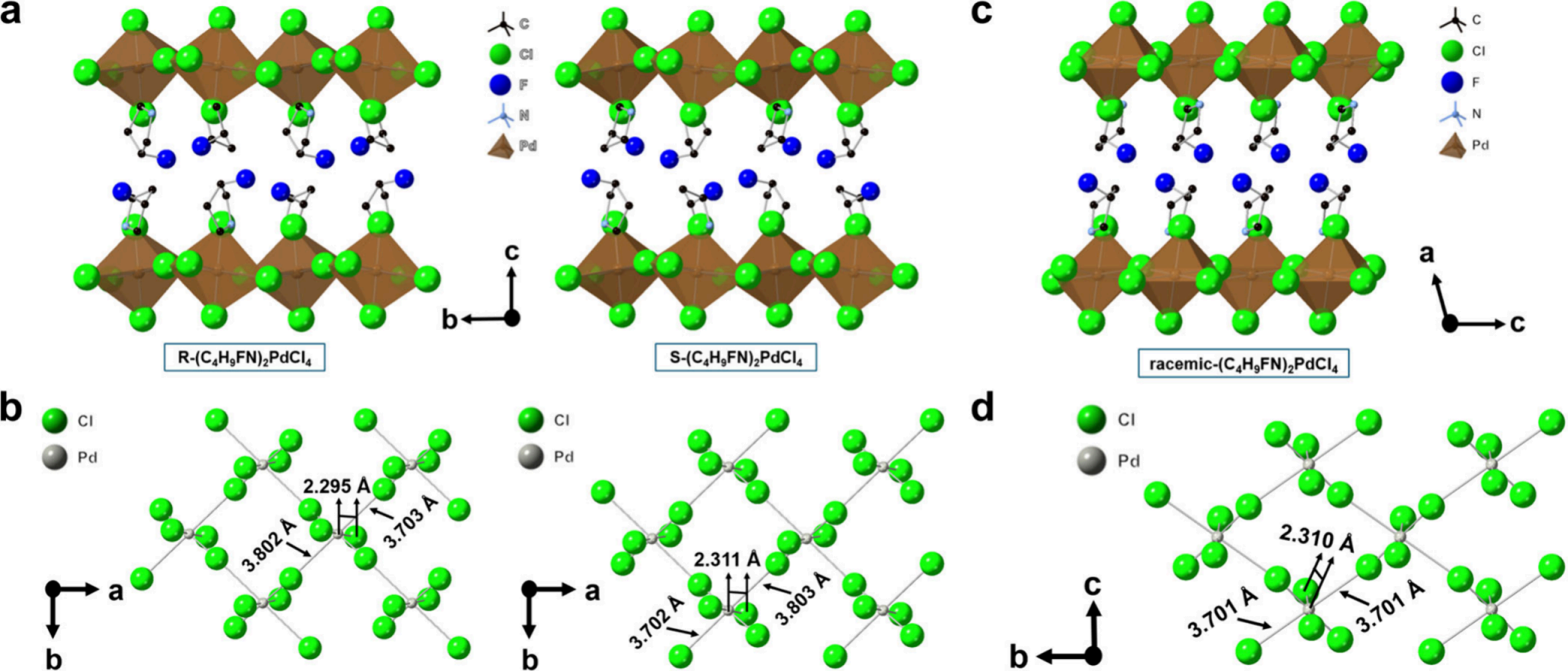
Crystal structures of (a and b) R- and S-(C_4_H_9_FN)_2_PdCl_4_ and (c and d) racemic (C_4_H_9_FN)_2_PdCl_4_. H atoms have been omitted for the sake of clarity.

**Table 2:** Single-Crystal Data and Structural Refinement Parameters for (C_4_H_9_FN)_2_PdCl_4_

	R-(C_4_H_9_FN)_2_PdCl_4_	S-(C_4_H_9_FN)_2_PdCl_4_	racemic (C_4_H_9_FN)_2_PdCl_4_
formula weight (g/mol)	428.44	428.44	428.44
temperature (K)	300	300	300
wavelength (Å)	0.71073	0.71073	1.54178
crystal system	monoclinic	monoclinic	monoclinic
space group	*P*2_1_ (#4)	*P*2_1_ (#4)	*P*2_1_/*c* (#14)
*Z*	2	2	2
unit cell parameters	*a* = 9.0441(2) Å	*a* = 9.0507(2) Å	*a* = 10.7982(2) Å
	*b* = 7.9200(2) Å	*b* = 7.9264(2) Å	*b* = 9.2064(2) Å
	*c* = 10.8166(3) Å	*c* = 10.8233(2) Å	*c* = 7.5428(2) Å
	β = 108.9920(10)°	β = 108.9870(10)°	β = 105.4380(10)°
volume (Å^3^)	732.61(3)	734.21(3)	722.79(3)
volume (Å^3^)/*Z*	366.31(2)	367.11(2)	361.40(2)
density (g/cm^3^)	1.942	1.938	1.969
absorption coefficient (μ) (mm^–1^)	1.998	1.993	17.220
θ_min_ – θ_max_ (deg)	4.764–60.992	4.76–61.062	12.836–140.03
no. of reflections collected	25 372	20 988	15 070
no. of independent reflections	4441	4458	1368
*R* ^*a*^ indices (*I* > 2σ(*I*))	*R*_1_ = 0.0325	*R*_1_ = 0.0250	*R*_1_ = 0.0162
	*wR*_2_ = 0.0640	*wR*_2_ = 0.0587	*wR*_2_ = 0.0402
goodness of fit on *F* ^2^	1.097	1.078	1.094
largest difference peak/hole (e^–^/Å^3^)	0.45/–0.84	0.49/–0.46	0.39/–0.34
Flack parameter	–0.01(2)	0.009(15)	–

Figure 4 and Figures S6–S8 present the crystal structure of R/S/racemic-(C_5_H_12_N)_2_PdCl_4_, which also are Ruddlesden–Popper 2D perovskites like R/S/racemic-(C_4_H_9_FN)_2_PdCl_4_ and (C_4_H_10_N)_2_PdCl_4_. Similar to R/S/racemic-(C_4_H_9_FN)_2_PdCl_4_, R/S-(C_5_H_12_N)_2_PdCl_4_ also crystallizes in the monoclinic *P*2_1_ (#4) space group and racemic (C_5_H_12_N)_2_PdCl_4_ crystallizes in the monoclinic *P*2_1_/*c* (#14) space group (Table 3), and the choice of space group is supported by the systematic absences, with precession images shown Figures S9 and 10. The short Pd–Cl bond distance within a single PdCl_6_ pseudo-octahedron is again 2.30–2.31 Å (Figure 4). The long Pd–Cl contacts in R/S/racemic-(C_5_H_12_N)_2_PdCl_4_ (∼4.1 Å) are notably longer than the long Pd–Cl contacts in (C_4_H_9_FN)_2_PdCl_4_. However, the difference between the two long Pd–Cl contacts in R/S-(C_5_H_12_N)_2_PdCl_4_ (0.084 Å) is slightly smaller than the difference between the two long Pd–Cl contacts in R/S-(C_4_H_9_FN)_2_PdCl_4_ (0.10 Å) (Figures 3 and 4). Unlike in racemic (C_4_H_9_FN)_2_PdCl_4_, where the R and S cations occupy distinct positions within the crystal structure, the cation in racemic (C_5_H_12_N)_2_PdCl_4_ forms a solid solution, where there is a 50% chance that either R- or S-(C_5_H_12_N)^+^ occupies a given cation site (Figure 4c). While it is also possible that there is an unresolved superstructure, we consider this unlikely given that the unit cell is similar in size to racemic (C_4_H_9_FN)_2_PdCl_4_, where the R and S cations order, and the precession images do not support the presence of an ordered superstructure (Figure S11). Interestingly, Wallach’s rule does not hold for (C_5_H_12_N)_2_PdCl_4_ because the racemic structure is less dense than its enantiomerically pure counterparts (Table 3; 1.699 g/cm^3^ for R-(C_5_H_12_N)_2_PdCl_4_, 1.700 g/cm^3^ for S-(C_5_H_12_N)_2_PdCl_4_, and 1.694 g/cm^3^ for racemic (C_5_H_12_N)_2_PdCl_4_), which is further evidence that the R and S cations form a solid solution as the cationic disorder does not allow the racemic compound to pack more densely than in the enantiomerically pure materials. We hypothesize that the reason racemic (C_5_H_12_N)_2_PdCl_4_ forms a cationic solid solution whereas the R and S cations in racemic (C_4_H_9_FN)_2_PdCl_4_ are ordered is that fluorine’s extremely high electronegativity compared to methyl greatly increases the effect of hydrogen bonding and other electrostatic interactions between the cations themselves and between cations and the inorganic framework. The strength of these interactions in racemic (C_5_H_12_N)_2_PdCl_4_ is too small such that any reduction in energy from the ordering of R and S cations cannot overcome the increase in entropy that results from the formation of a solid solution.^34^

**Figure 4:**
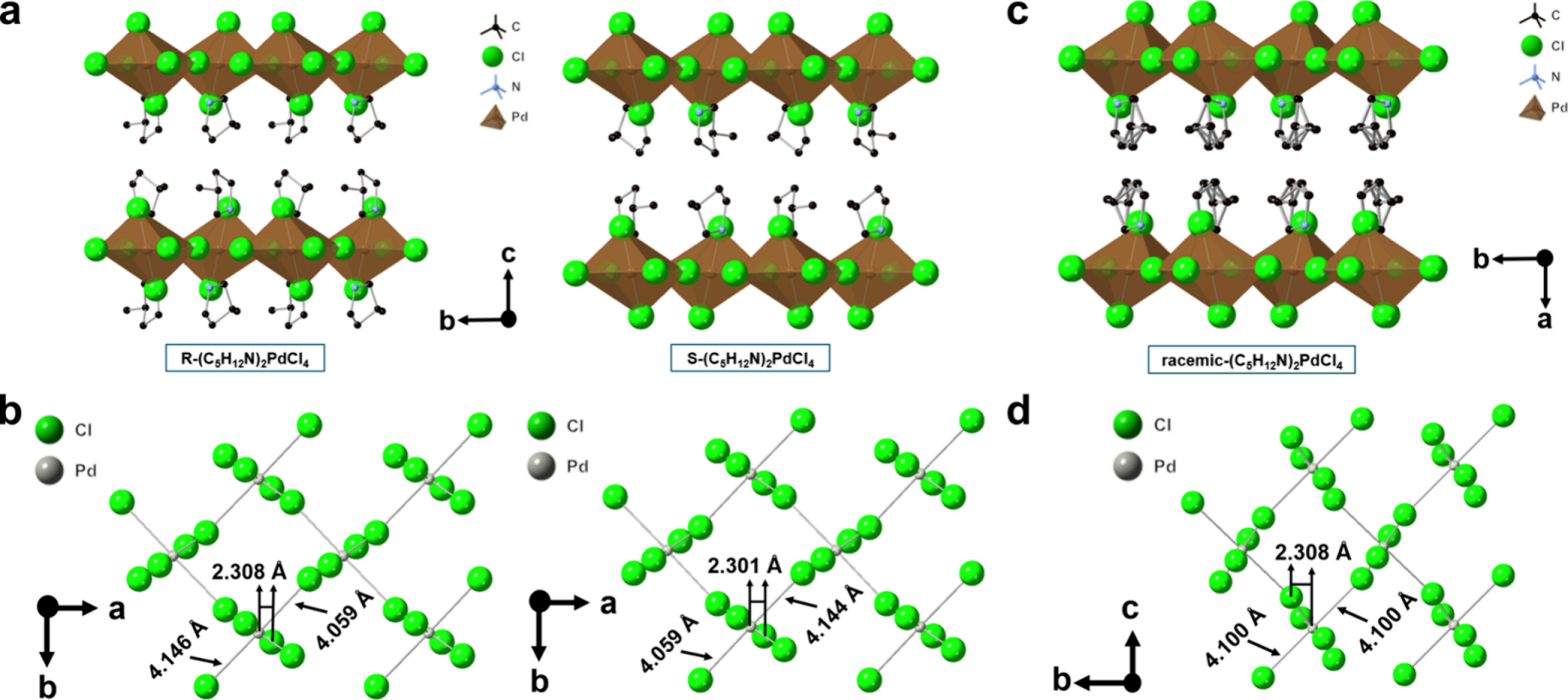
Crystal structures of (a and b) R- and S-(C_5_H_12_N)_2_PdCl_4_ and (c and d) racemic solid solution (C_5_H_12_N)_2_PdCl_4_. H atoms have been omitted for the sake of clarity.

**Table 3:** Single-Crystal Data and Structural Refinement Parameters for (C_5_H_12_N)_2_PdCl_4_

	R-(C_5_H_12_N)_2_PdCl_4_	S-(C_5_H_12_N)_2_PdCl_4_	racemic (C_5_H_12_N)_2_PdCl_4_
formula weight (g/mol)	420.52	420.52	420.52
temperature (K)	300	300	300
wavelength (Å)	0.71073	0.71073	0.71073
crystal system	monoclinic	monoclinic	monoclinic
space group	*P*2_1_ (#4)	*P*2_1_ (#4)	*P*2_1_/*c* (#14)
*Z*	2	2	2
unit cell parameters	*a* = 9.8639(2) Å	*a* = 9.8633(2) Å	*a* = 11.3256(7) Å
	*b* = 8.08190(10) Å	*b* = 8.08020(10) Å	*b* = 8.1168(5) Å
	*c* = 11.3229(2) Å	*c* = 11.3212(2) Å	*c* = 9.8359(6) Å
	β = 114.4060(10)°	β = 114.4050(10)°	β = 114.2430(10)°
volume (Å^3^)	821.99(3)	821.65(3)	824.45(9)
volume (Å^3^)/*Z*	411.00(2)	410.83(2)	412.23(5)
density (g/cm^3^)	1.699	1.700	1.694
absorption coefficient (μ) (mm^–1^)	1.762	1.763	1.757
θ_min_ – θ_max_ (deg)	4.534–72.888	4.534–72.84	6.384–72.73
no. of reflections collected	98 260	81 182	35 642
no. of independent reflections	8031	7999	3966
*R* ^*a*^ indices (*I* > 2σ(*I*))	*R*_1_ = 0.0266	*R*_1_ = 0.0312	*R*_1_ = 0.0230
	*wR*_2_ = 0.0794	*wR*_2_ = 0.0656	*wR*_2_ = 0.0599
goodness of fit on *F* ^2^	1.043	1.083	1.052
largest difference peak/hole (e^–^/Å^3^)	0.50/–0.60	0.46/–0.53	0.57/–0.43
Flack parameter	0.024(10)	0.036(16)	–

Overall, we observe that the organic cation size is directly related to the determined unit cell volume, where racemic (C_5_H_12_N)_2_PdCl_4_ has the largest normalized cell volume (*V*/*Z*) of 412.23(5) Å^3^ while achiral (C_4_H_10_N)_2_PdCl_4_ shows the smallest *V*/*Z* of 358.17(1) Å^3^ (Tables 1–3). A similar trend was previously observed in (PEA)_2_PbI_4_ (PEA = phenethylammonium) and derivatives containing functionalized PEA cations,^35,36^ where longer cations resulted in larger unit cells. In addition, it was found that (4-MePEA)_2_PbI_4_ exhibits cationic disorder, whereas (PEA)_2_PbI_4_ and (4-XPEA)_2_PbI_4_ (X = F, Cl, or Br) have ordered cation arrangements,^35^ which is similar to what we find here where the cations in racemic (C_5_H_12_N)_2_PdCl_4_ form a solid solution. Another trend that we identify is that the length of the long Pd–Cl contact correlates with the size of the organic cation, whereas the length of the true Pd–Cl bonds does not change. Since cation (C_4_H_10_N)^+^ is the smallest among (C_4_H_10_N)^+^, (C_5_H_12_N)^+^, and (C_4_H_9_FN)^+^, the long Pd–Cl contact is also the shortest in (C_4_H_10_N)_2_PdCl_4_, with the exception being that the long Pd–Cl contact in racemic (C_4_H_9_FN)_2_PdCl_4_ is slightly shorter than the long Pd–Cl contact in (C_4_H_10_N)_2_PdCl_4_.

In addition to using single-crystal X-ray diffraction, we also characterized the materials using powder X-ray diffraction (PXRD). The measured PXRD patterns are shown in Figure S12. As expected, the experimental PXRD pattern matches the pattern calculated from the crystal structure, indicating that the bulk materials are phase pure. We also studied the materials’ stability against ambient air exposure. The results are presented in Figure S13. The diffraction patterns show that the materials do not change phase over 2–4 weeks of air exposure, although a decrease in scattering intensity is observed for all materials.

To better understand how structural trends correlate with electronic properties, we performed DFT calculations on the experimentally determined crystal structures using WIEN2k (see the Computational Methods).^37,38^ Figures 5–7 show the computed band structures and density of states (DOS) of achiral (C_4_H_10_N)_2_PdCl_4_ (Figure 5), R/S/racemic-(C_4_H_9_FN)_2_PdCl_4_ (Figure 6), and R/S/racemic-(C_5_H_12_N)_2_PdCl_4_ (Figure 7). As shown by the band structure plots, the lowest unoccupied molecular orbitals (LUMOs) are not dispersive, indicating that these orbitals are highly localized on each Pd^2+^ atom. There is some dispersion in the highest occupied molecular orbitals (HOMOs) for all materials that reflects the four equivalent Pd–Cl bonds in a square planar geometry. The highly localized orbitals indicate that these Pd­(II) compounds are electronically quasi-zero-dimensional as there is little interaction between Pd^2+^ atoms within the structure.^39^

**Figure 5:**
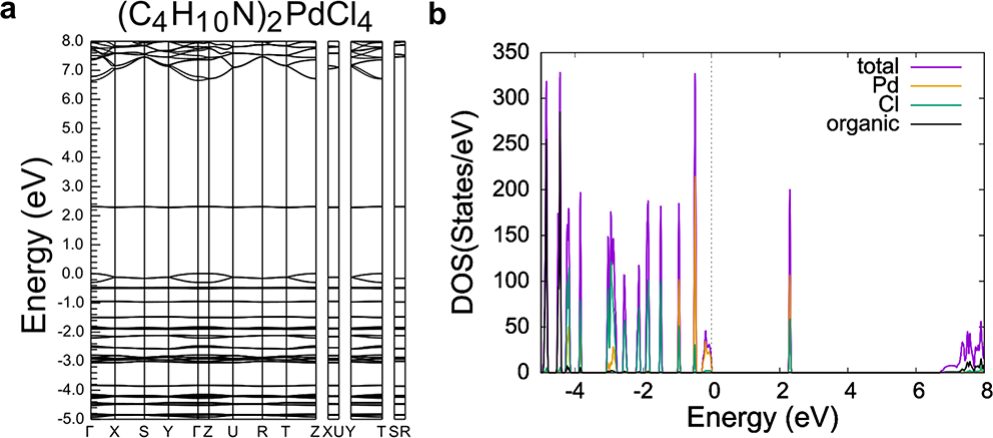
(a) Band structure and (b) density of states plots of achiral (C_4_H_10_N)_2_PdCl_4_. The Fermi energy is set to 0 eV.

**Figure 6:**
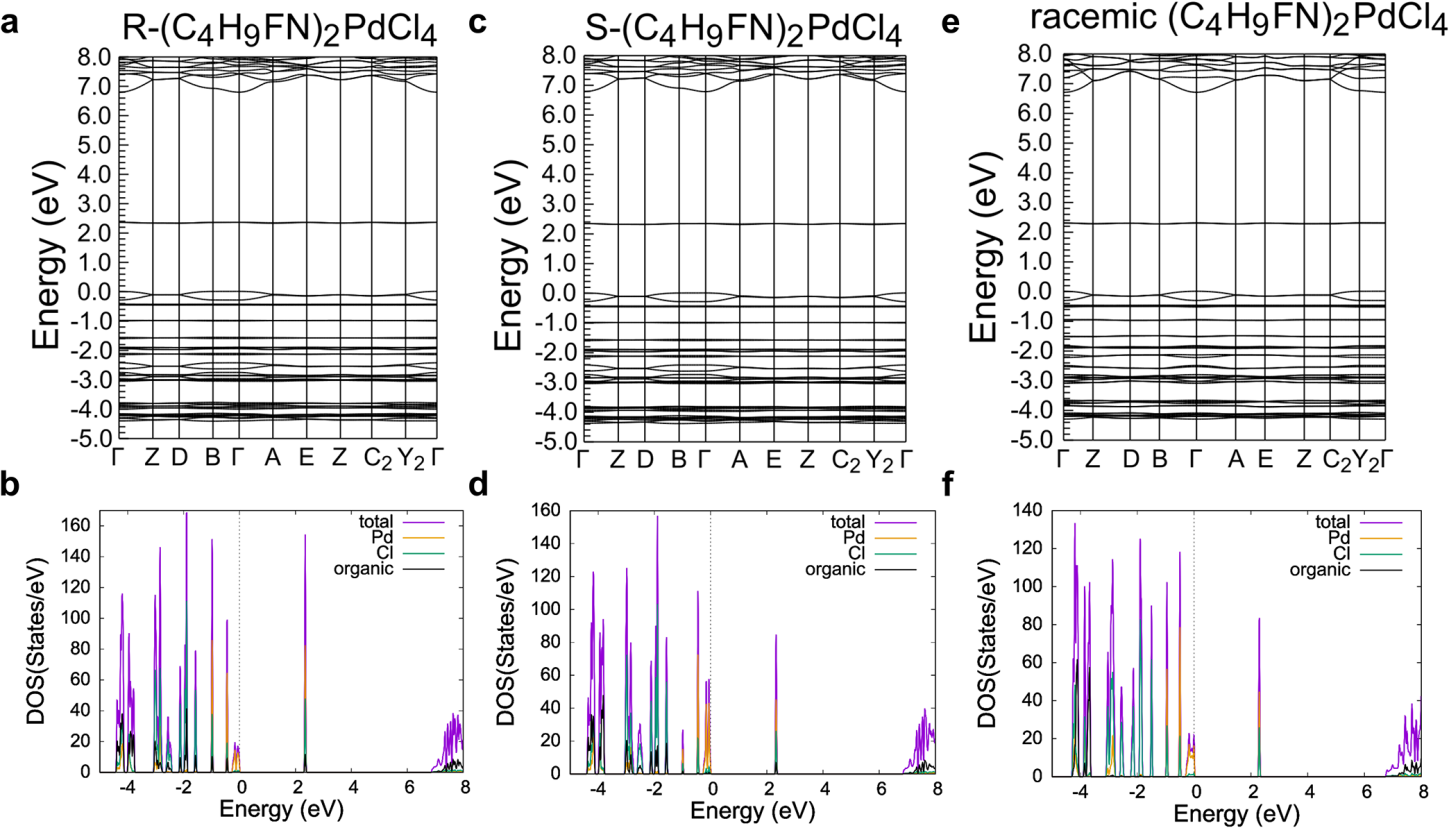
Band structure and density of states plots of (a and b) R-(C_4_H_9_FN)_2_PdCl_4_, (c and d) S-(C_4_H_9_FN)_2_PdCl_4_, and (e and f) racemic (C_4_H_9_FN)_2_PdCl_4_. The Fermi energy is set to 0 eV.

**Figure 7:**
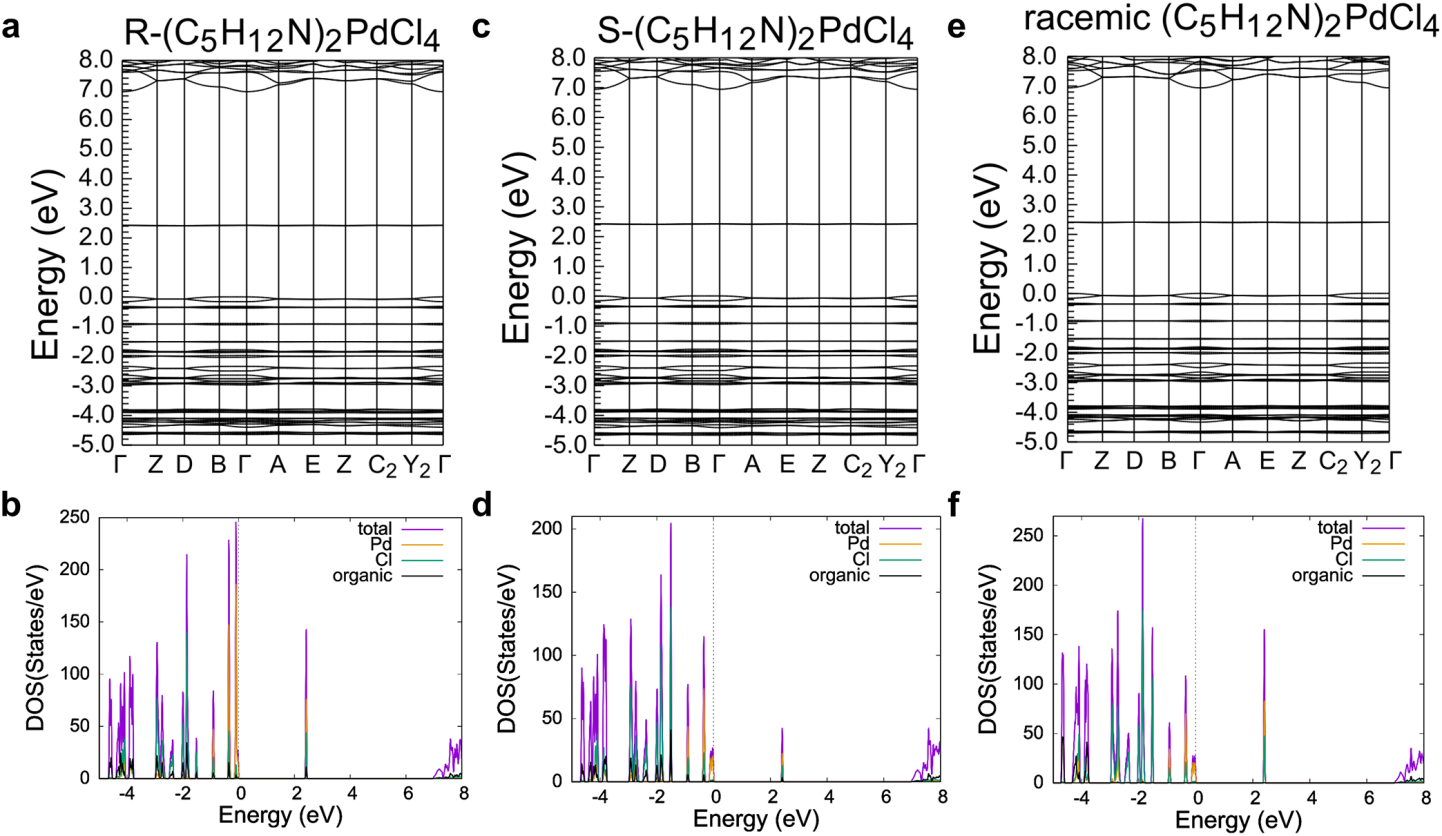
Band structure and density of states plots of (a and b) R-(C_5_H_12_N)_2_PdCl_4_, (c and d) S-(C_5_H_12_N)_2_PdCl_4_, and (e and f) racemic solid solution (C_5_H_12_N)_2_PdCl_4_. The Fermi energy is set to 0 eV.

For the synthesized achiral Pd­(II) compounds, the HOMOs and LUMOs are dominated by Pd and Cl states with little contribution from the organic cations, which is typical for organic–inorganic hybrid materials such as 0D (C_8_H_20_P)­InBr_4_ (where (C_8_H_20_P)^+^ = tetraethylphosphonium), 2D (C_5_H_10_F_2_N)­CuBr_2_ ((C_5_H_10_F_2_N)^+^ = 4,4-difluoropiperidinium), and 2D (C_6_H_18_N_2_)­PbI_4_ ((C_6_H_18_N_2_)^2+^ = hexane-1,6-diaminium), where electrostatic forces couple the organic and inorganic frameworks.^40–43^ In the synthesized chiral materials, however, the HOMO and LUMO (and all bands, for that matter) have a small contribution from the organic cations, which is consistent with a recent DFT study that found that in chiral organic–inorganic hybrid materials, the chiral organic cations polarize the electronic orbitals of the metal halide framework; this effect is not present in the synthesized achiral materials, including those containing a racemic mixture of cations, because these materials crystallize in centrosymmetric space groups.^44^

The calculated HOMO–LUMO (or, alternately, valence band maximum (VBM) and conduction band minimum (CBM)) gap for the synthesized Pd­(II) compounds is listed in Table 4 and is shown in Figure 8. As one can observe, the HOMO–LUMO gap scales nearly linearly (dashed line in Figure 8; *R* ^2^ = 0.97) with the length of the longer of the two Pd–Cl contacts as the smallest gap is for racemic (C_4_H_9_FN)_2_PdCl_4_ while the largest is for R- and S-(C_5_H_12_N)_2_PdCl_4_, demonstrating that there is reduced electronic coupling as the length of the long contacts increases.

**Table 4:** HOMO–LUMO Gaps from DFT Calculations and Lengths of the Long Pd–Cl Contact

material	HOMO–LUMO gap (eV)	long Pd–Cl contact length(s) (Å)
(C_4_H_10_N)_2_PdCl_4_	2.288	3.7190(8)
R-(C_4_H_9_FN)_2_PdCl_4_	2.337	3.7032(18), 3.8019(18)
S-(C_4_H_9_FN)_2_PdCl_4_	2.320	3.7017(12), 3.8035(12)
racemic (C_4_H_9_FN)_2_PdCl_4_	2.282	3.7014(4)
R-(C_5_H_12_N)_2_PdCl_4_	2.413	4.0586(12), 4.1461(12)
S-(C_5_H_12_N)_2_PdCl_4_	2.414	4.0593(17), 4.1436(17)
racemic (C_5_H_12_N)_2_PdCl_4_	2.397	4.0999(4)

**Figure 8:**
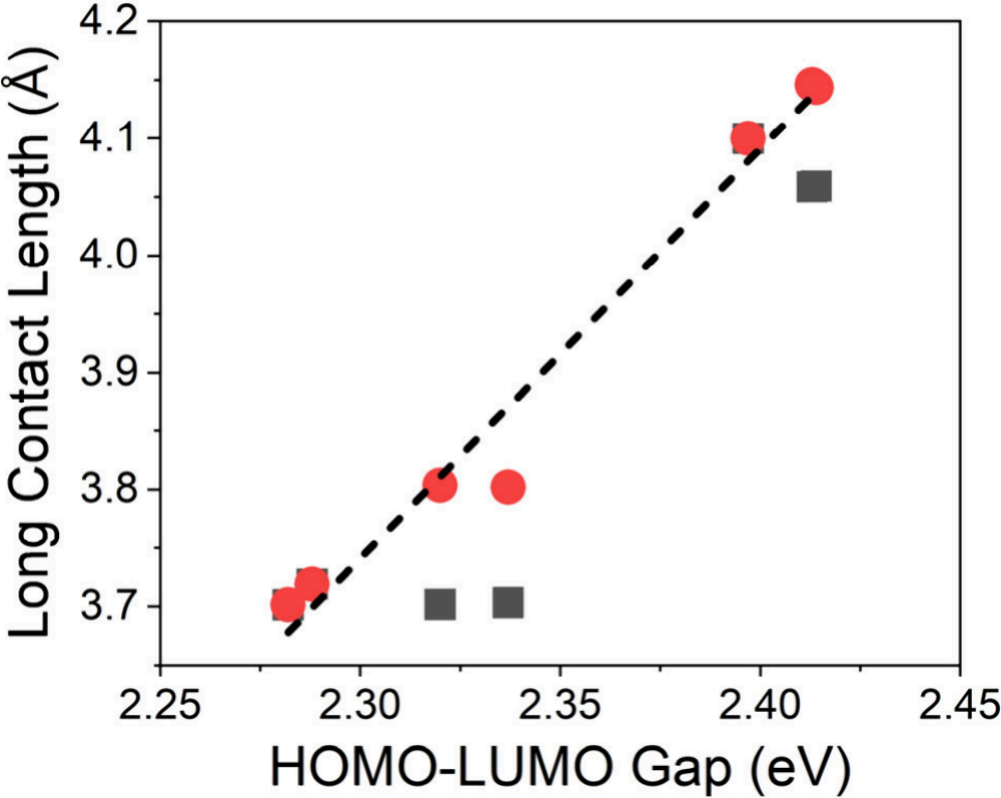
Plot of the HOMO–LUMO gap vs the length of the long Pd–Cl contact(s). A linear regression of the points in red (*R* ^2^ = 0.97) is shown as a dashed black line.

Finally, we studied the chiroptical properties of the (C_4_H_9_FN)_2_PdCl_4_ and (C_5_H_12_N)_2_PdCl_4_ thin films using CD spectroscopy. As expected, for racemic (C_4_H_9_FN)_2_PdCl_4_, no CD signal was observed, and R- and S-(C_4_H_9_FN)_2_PdCl_4_ present opposite CD signals (Figure 9). The obtained CD data are consistent with the collected absorbance spectra using a thin film (see Figure S14). CD measurements were also attempted for chiral (C_5_H_12_N)_2_PdCl_4_. However, for deposited R- and S-(C_5_H_12_N)_2_PdCl_4_ thin films, we were not able to observe any CD. The difference in CD strength for chiral (C_4_H_9_FN)_2_PdCl_4_ and (C_5_H_12_N)_2_PdCl_4_ is likely due to stronger interactions between the fluorinated organic cation and the Pd–Cl framework compared with the methylated cation, increasing the degree to which chirality is transferred from the chiral organic cation to the inorganic framework. Moreover, the calculated distortion indices^45^ for pseudo-octahedral coordinated Pd in R-(C_4_H_9_FN)_2_PdCl_4_ and R-(C_5_H_12_N)_2_PdCl_4_ are 0.231 and 0.275, respectively. The calculated value of the distortion index for R-(C_5_H_12_N)_2_PdCl_4_ is higher than that for R-(C_4_H_9_FN)_2_PdCl_4_ and is much higher than the distortion index calculated for R-(MBA)_2_PdCl_4_ (0.00246),^24^ which exhibits strong CD. This indicates that differences in the distorted Pd pseudo-octahedral geometry are not responsible for the difference in chiroptical properties between chiral (C_4_H_9_FN)_2_PdCl_4_ and chiral (C_5_H_12_N)_2_PdCl_4_ and that the distortion index is not a valid predictor of the CD signal in these materials. The lack of an observable CD signal in R/S-(C_5_H_12_N)_2_PdCl_4_ is consistent with the formation of a cationic solid solution in racemic (C_5_H_12_N)_2_PdCl_4_ as the poor chirality transfer from the organic to inorganic framework is indicative of weak interactions between organic cations and/or between the cations and the inorganic framework.^24,46^

**Figure 9:**
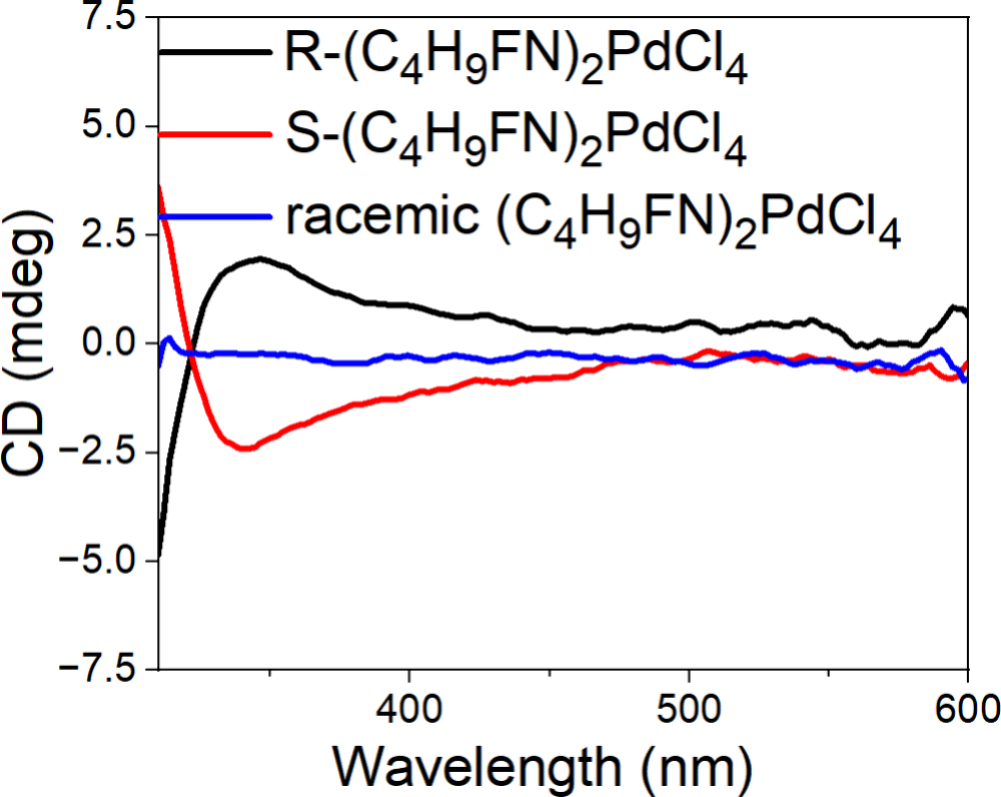
CD spectra of R/S-(C_4_H_9_FN)_2_PdCl_4_ and racemic (C_4_H_9_FN)_2_PdCl_4_ thin films.

## 3 Conclusion

To summarize, we report new palladium­(II) chloride materials (C_4_H_10_N)_2_PdCl_4_, (C_4_H_9_FN)_2_PdCl_4_, and (C_5_H_12_N)_2_PdCl_4_, which exhibit significant differences in their structural, electronic, and chiroptical behavior that originate from small changes to the organic cation’s structure. As the size of the cation increases ((C_5_H_12_N)^+^ > (C_4_H_9_FN)^+^ > (C_4_H_10_N)^+^), the normalized volume of the unit cell also increases. In addition, the length of the long Pd–Cl contact(s) in a single PdCl_6_ octahedron scales with the size of the cation, although this trend is less strong than the trend for the volume of the unit cell; the length of the four Pd–Cl bonds is nearly identical in all of these materials. Interestingly, unlike in racemic (C_4_H_9_FN)_2_PdCl_4_, where the R and S cations occupy distinct positions, the R and S cations in racemic (C_5_H_12_N)_2_PdCl_4_ form a solid solution where there is a 50% chance that either R- or S-(C_5_H_12_N)^+^ occupies a given cation site. Accordingly, Wallach’s density rule holds true for (C_4_H_9_FN)_2_PdCl_4_ but not (C_5_H_12_N)_2_PdCl_4_, indicating the importance of the cation in directing the properties of organic–inorganic hybrid metal halide materials. Band structure calculations show that the obtained Pd­(II) compounds have a molecular-like quasi-0D electronic structure, indicating that there is only weak coupling between Pd^2+^ ions. Furthermore, the calculated HOMO–LUMO gap is directly proportional to the length of the long Pd–Cl contact(s), and this trend matches what is expected from an analysis of the molecular orbitals of the PdCl_6_ octahedra. Lastly, R/S-(C_4_H_9_FN)_2_PdCl_4_ thin films show appreciable CD signals while R/S-(C_5_H_12_N)_2_PdCl_4_ thin films do not, indicating that the stronger interactions between the cations and the inorganic framework significantly increase the strength of the chiroptical properties. Our results emphasize the importance of the organic cation in tuning the structure and properties of chiral, racemic, and achiral 2D palladium chloride materials.

## 4 Experimental Methods

### 4.1 Crystal Growth

For the crystal growth of R/S-(C_4_H_9_FN)_2_PdCl_4_, 1 mmol of PdCl_2_ (99.999%, Fisher Scientific) and 1 mmol of R/S-3-fluoropyrrolidine hydrochloride (Ossila and Ambeed, Inc.) were dissolved in 4 mL of HCl acid. The clear solution was then left in air for slow evaporation. After a few days of continuous crystal growth, R/S-(C_4_H_9_FN)_2_PdCl_4_ crystals were harvested, dried, and stored in a desiccator. For the synthesis of racemic (C_4_H_9_FN)_2_PdCl_4_, 1 mmol of PdCl_2_, 1 mmol of R-3-fluoropyrrolidine hydrochloride, and 1 mmol of S-3-fluoropyrrolidine hydrochloride were dissolved in 8 mL of HCl acid. The clear solution was then left in air for slow evaporation.

For the crystal growth of R/S-(C_5_H_12_N)_2_PdCl_4_, 1 mmol of PdCl_2_ (99.999%, Fisher Scientific) and 1 mmol of R/S-3-methylpyrrolidine hydrochloride (ChemScene and Ambeed, Inc.) were dissolved in 4 mL of HCl acid. The clear solution was then left in air for slow evaporation. After a few days of continuous crystal growth, R/S-(C_5_H_12_N)_2_PdCl_4_ crystals were harvested, dried, and stored in a desiccator. For the synthesis of racemic (C_5_H_12_N)_2_PdCl_4_, 1 mmol of PdCl_2_, 0.5 mmol of R-3-methylpyrrolidine hydrochloride, and 0.5 mmol of S-3-methylpyrrolidine hydrochloride were dissolved in 10 mL of HCl acid. The solution was then filtered using 0.22 μm PTFE filters (VWR, Inc.). The filtered solution was then left in air for slow evaporation.

For the crystal growth of (C_4_H_10_N)_2_PdCl_4_, 1 mmol of PdCl_2_ (99.999%, Fisher Scientific) and 1 mmol of pyrrolidine hydrochloride (ChemScene, Inc.) were dissolved in 4 mL of HCl acid. The clear solution was then left in air for slow evaporation. After a few days of continuous crystal growth, (C_4_H_10_N)_2_PdCl_4_ crystals were harvested, dried, and stored in a desiccator.

### 4.2 X-ray Diffraction (XRD) Measurements

Single-crystal X-ray diffraction data were collected at room temperature using a Bruker D8 Venture diffractometer equipped with a Photon III 14 detector using Cu Kα radiation or a Bruker D8 Quest diffractometer equipped with a Photon III 7 detector using Mo Kα radiation. The initial solution was determined using ShelXT,^47^ and the structure was refined using ShelXL^48^ with the Olex2 GUI.^49^ The crystal structures were deposited into the Cambridge Crystallographic Data Centre database with deposition numbers 2477919–2477925. For powder X-ray diffraction (PXRD) measurements, crystals were first ground using a mortar and pestle, and PXRD patterns were then taken using a Panalytical Aeris X-ray diffractometer. To monitor the sample’s stability against ambient air, powders were left in air and periodic PXRD measurements were taken over time to monitor the PXRD pattern change.

### 4.3 Thin Film Deposition, Circular Dichroism (CD), and Absorbance Measurement

To perform CD measurements, (C_4_H_9_FN)_2_PdCl_4_ and (C_5_H_12_N)_2_PdCl_4_ thin films were first made using a glass substrate. The glass substrate is 25 mm × 25 mm and was cleaned using a cleaning solution (prepared by mixing 5% Hellmanex III with 95% deionized water), followed by ethanol, diluted HCl acid, and acetone (10 min for each). To prepare R/S-(C_4_H_9_FN)_2_PdCl_4_ films, 30 mg of R/S-(C_4_H_9_FN)_2_PdCl_4_ crystals was dissolved in 0.6 mL of DMF. The solution was then spun coated on the glass substrate (spin speed of 2000 rpm and spin time of 30 s). The deposited film was then annealed using a hot plate at 70 °C for 15 min. To prepare the racemic (C_4_H_9_FN)_2_PdCl_4_ film, 15 mg of racemic crystals was dissolved using 1.2 mL of DMF. The racemic (C_4_H_9_FN)_2_PdCl_4_ film was then made and annealed at 70 °C for 15 min.

For R/S-(C_5_H_12_N)_2_PdCl_4_ films, 30 mg of R/S-(C_5_H_12_N)_2_PdCl_4_ crystals was first dissolved in 0.2 mL of DMF. The solution was then spin coated on the glass substrate (spin speed of 2000 rpm and spin time of 30 s). The deposited film was then annealed using a hot plate at 70 °C for 15 min.

CD measurements were performed for the deposited (C_4_H_9_FN)_2_PdCl_4_ and (C_5_H_12_N)_2_PdCl_4_ thin films by using the OLIS DSM 1000 CD instrument. CD data were acquired and processed using OLIS GlobalWorks. Absorbance data were collected on thin films using an Agilent Cary 50 UV–vis spectrometer.

## 5 Computational Methods

DFT calculations were performed using version 24.1 of the WIEN2k software package^37,38^ linked with version 2024.03.001 of the ELPA library.^50^ All calculations were performed on experimental crystal structures without geometry optimization. Initial calculations were performed using the GGA-PBE functional^51^ with energy and charge convergence thresholds of 0.0001 Ry and 0.0001 *e*, respectively. Subsequently, we used the local modified Becke–Johnson (mBJ) exchange-correlation potential^52^ for the density-of-states and band structure calculations because of its high accuracy for band gap calculations. We used mBJ parameters from reference,^53^ a Wigner–Seitz radius of 7 Bohr, and a smearing parameter of 3.78 Bohr. When using the traditional mBJ exchange potential, our calculation did not converge, which we hypothesize is caused by a large variation in the local charge density over the unit cell because of the presence of both organic and inorganic components in our materials. For racemic (C_5_H_12_N)_2_PdCl_4_, disorder was removed for DFT calculations, and the R and S cations were ordered like in racemic (C_4_H_9_FN)_2_PdCl_4_. *K*-paths for band structure plots were generated using SeeK-path.^54^

## Supplementary Material



## Notes

Crystal photograph, crystal structure thermal ellipsoid plots, precession images, powder X-ray diffraction (PXRD) pattern, and absorbance data (PDF)
