# Supplementary material for: Role of the Organic Cation in 2D Chiral Hybrid Palladium Chloride Materials
Source: Inorg Chem. 2025 Nov 10;64(46):22911–20. doi: 10.1021/acs.inorgchem.5c04105 (PMC12648662; doi:10.1021/acs.inorgchem.5c04105)
Supplement: Supplementary file 1 [file ic5c04105_si_001.pdf]

Supporting Information

Role of the Organic Cation in 2D Chiral Hybrid Palladium Chloride Materials

Zheng Zhang, Daniel B. Straus*
Department of Chemistry, Tulane University, New Orleans, LA, 70118 USA
*Email: dstraus@tulane.edu

1. Crystal Photograph

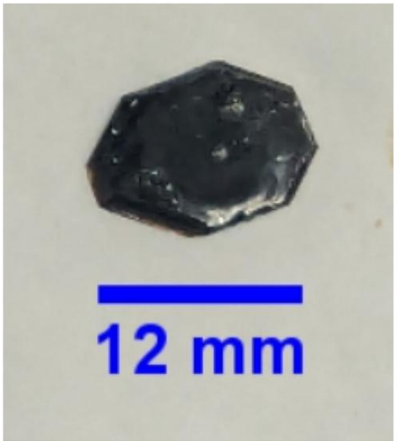

Figure S1. A representative photograph of an as-grown R-$(C_4H_9FN)_2PdCl_4$ crystal.

2. Crystal Structure Thermal Ellipsoid Plots

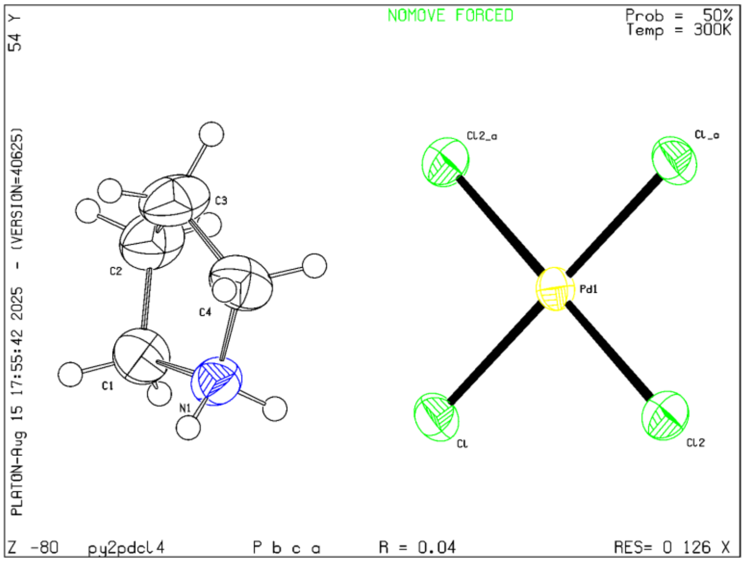

Figure S2. Depiction of asymmetric unit of crystal structure of achiral $(C_4H_{10}N)_2PdCl_4$ with atoms represented as 50% probability thermal ellipsoids.

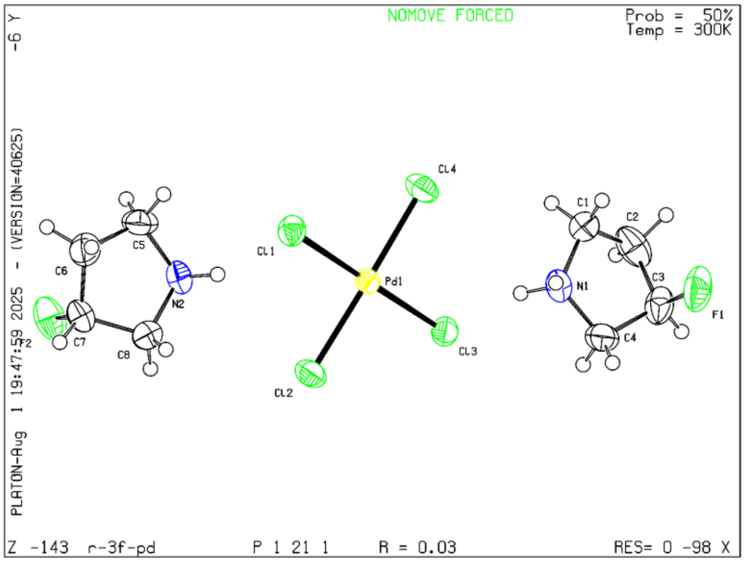

Figure S3. Depiction of asymmetric unit of crystal structure of R-$(C_4H_9FN)_2PdCl_4$ with atoms represented as 50% probability thermal ellipsoids.

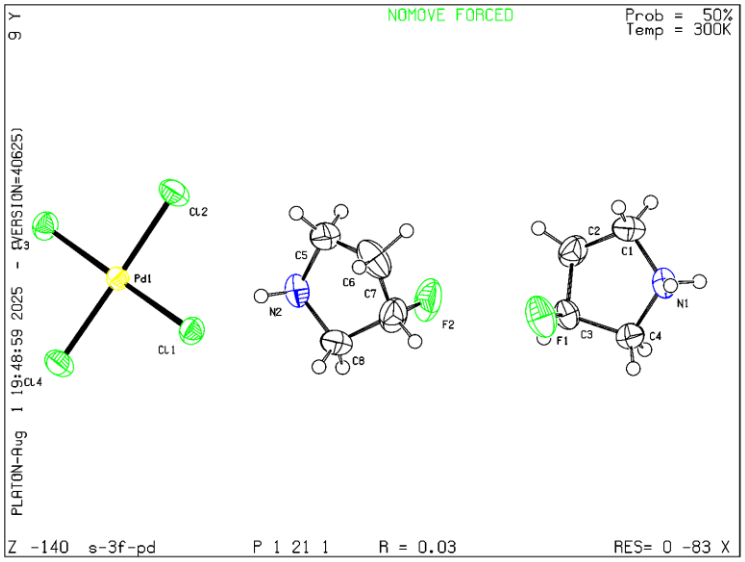

Figure S4. Depiction of asymmetric unit of crystal structure of S-$(C_4H_9FN)_2PdCl_4$ with atoms represented as 50% probability thermal ellipsoids.

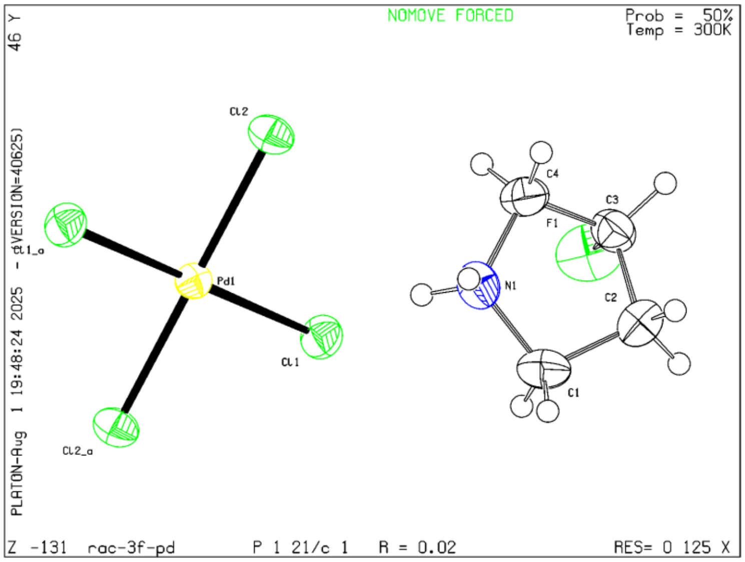

Figure S5. Depiction of asymmetric unit of crystal structure of racemic $(C_4H_9FN)_2PdCl_4$ with atoms represented as 50% probability thermal ellipsoids.

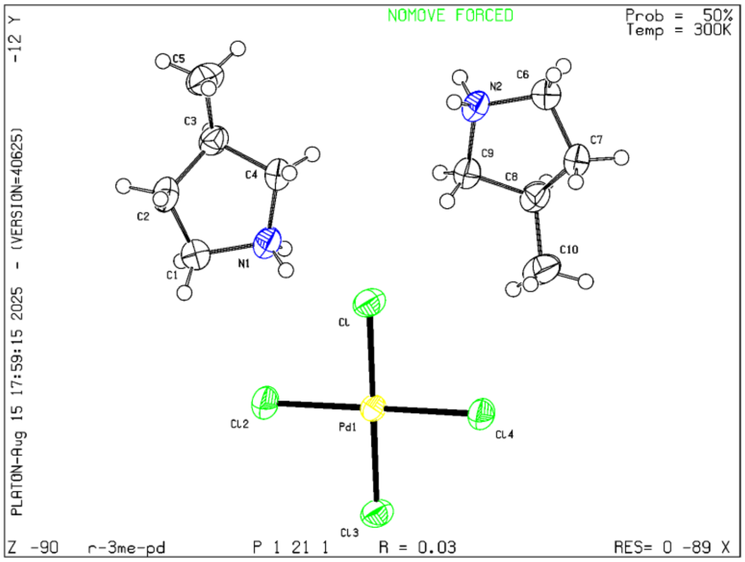

Figure S6. Depiction of asymmetric unit of crystal structure of R-$(C_5H_{12}N)_2PdCl_4$ with atoms represented as 50% probability thermal ellipsoids.

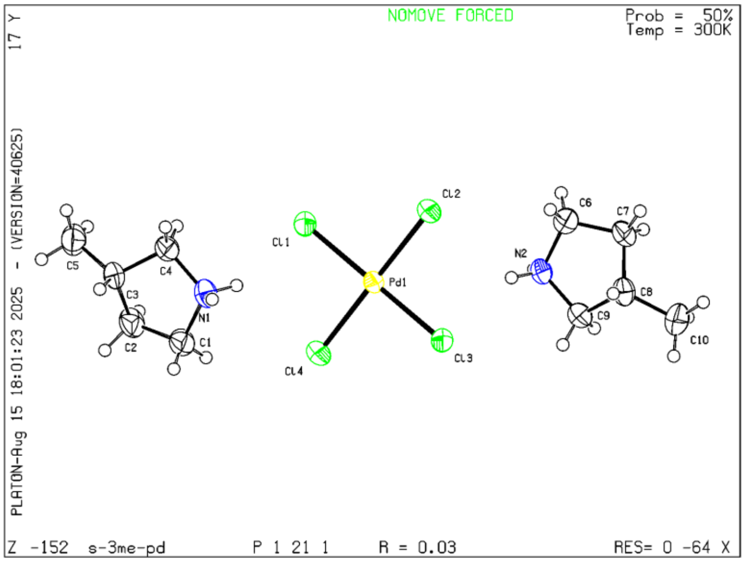

Figure S7. Depiction of asymmetric unit of crystal structure of S-$(C_5H_{12}N)_2PdCl_4$ with atoms represented as 50% probability thermal ellipsoids.

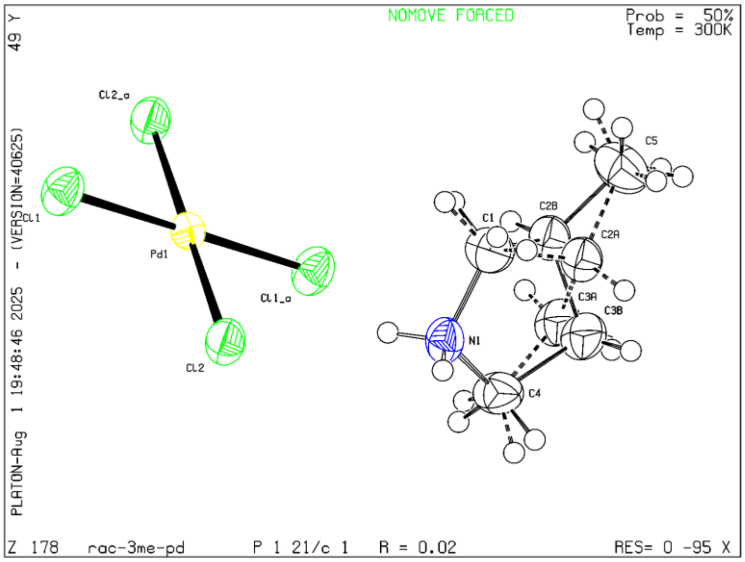

Figure S8. Depiction of asymmetric unit of crystal structure of racemic $(C_5H_{12}N)_2PdCl_4$ with atoms represented as 50% probability thermal ellipsoids.

3. Precession Images

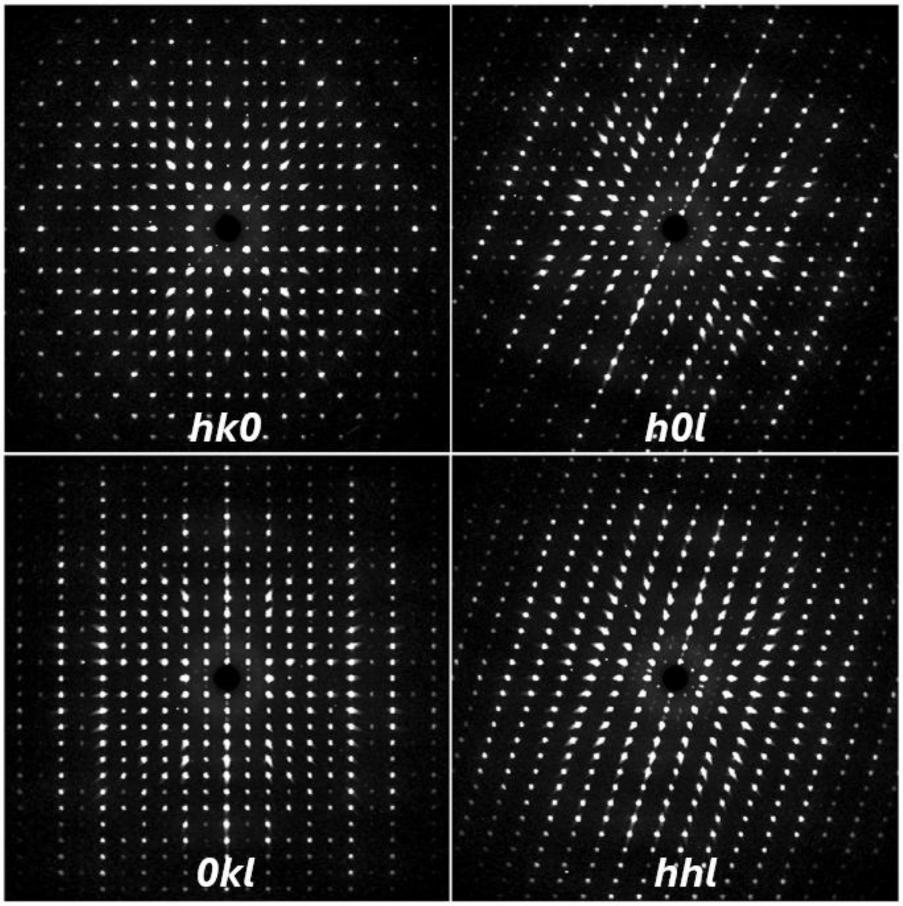

Figure S9. Single crystal X-ray diffraction precession image generated for R-$(C_5H_{12}N)_2PdCl_4$.

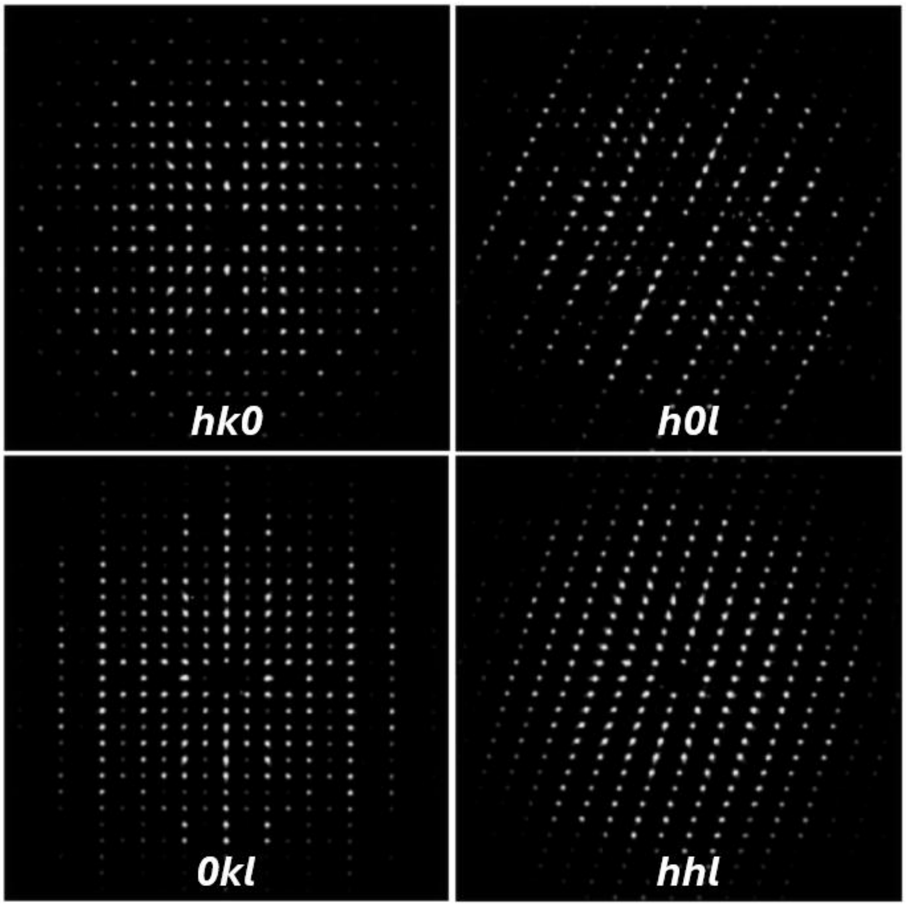

Figure S10. Single crystal X-ray diffraction precession image generated for S-$(C_5H_{12}N)_2PdCl_4$.

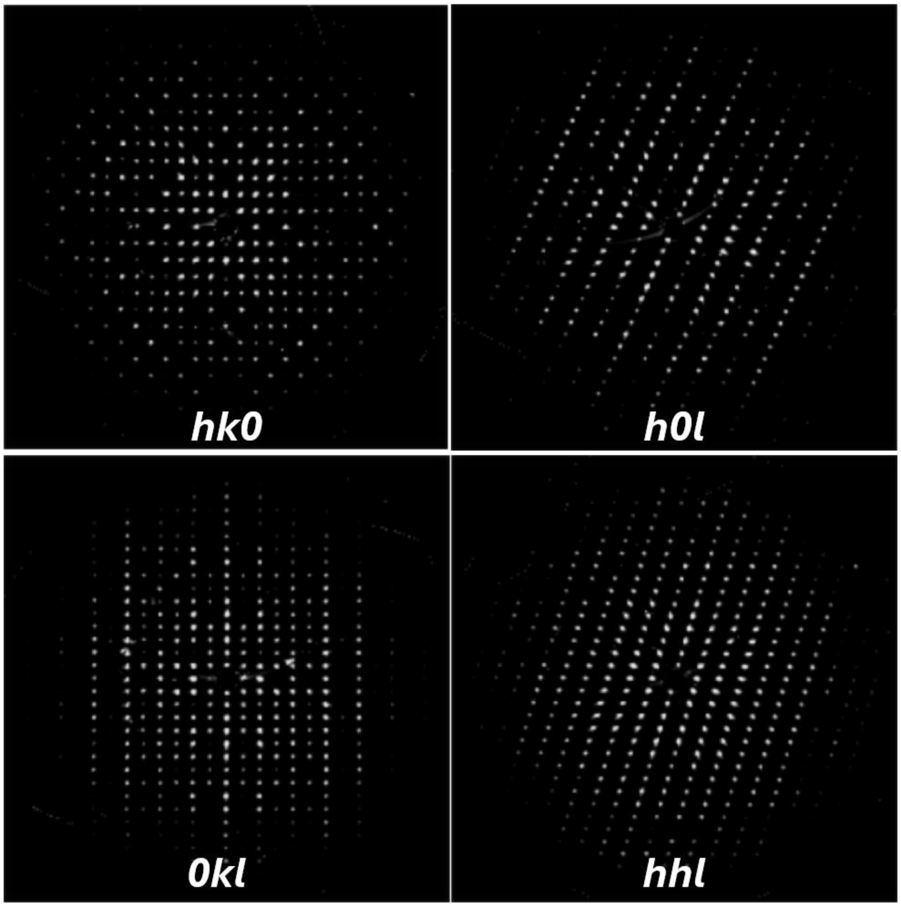

Figure S11. Single crystal X-ray diffraction precession image generated for racemic $(C_5H_{12}N)_2PdCl_4$.

4. Powder X-Ray Diffraction (PXRD) Pattern

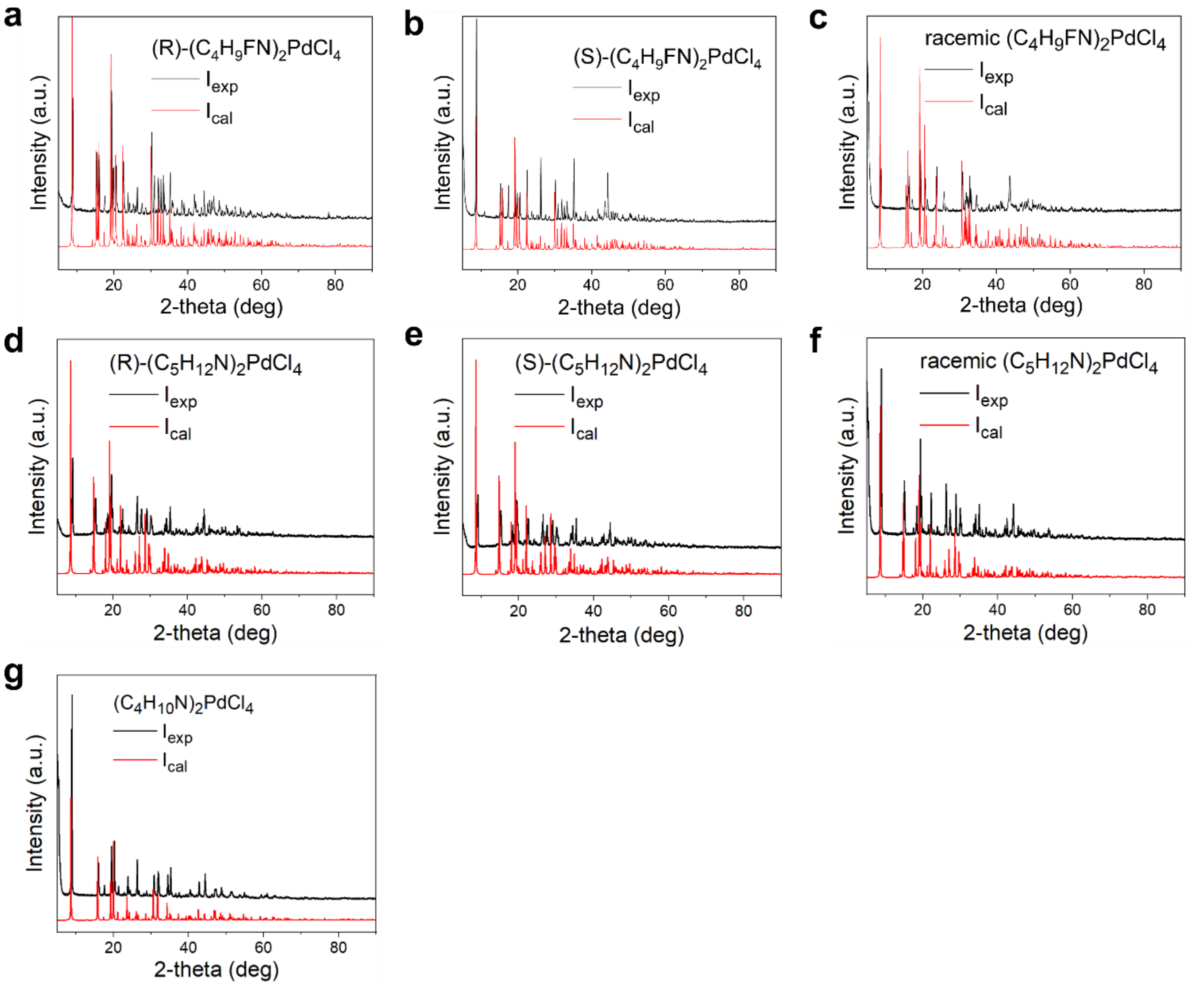

Figure S12. Experimental (I_{exp}) and calculated (I_{cal}) PXRD pattern of **(a)** R-$(C_4H_9FN)_2PdCl_4$, **(b)** S-$(C_4H_9FN)_2PdCl_4$, **(c)** racemic $(C_4H_9FN)_2PdCl_4$, **(d)** R-$(C_5H_{12}N)_2PdCl_4$, **(e)** S-$(C_5H_{12}N)_2PdCl_4$, **(f)** racemic solid solution $(C_5H_{12}N)_2PdCl_4$, **(g)** achiral $(C_4H_{10}N)_2PdCl_4$.

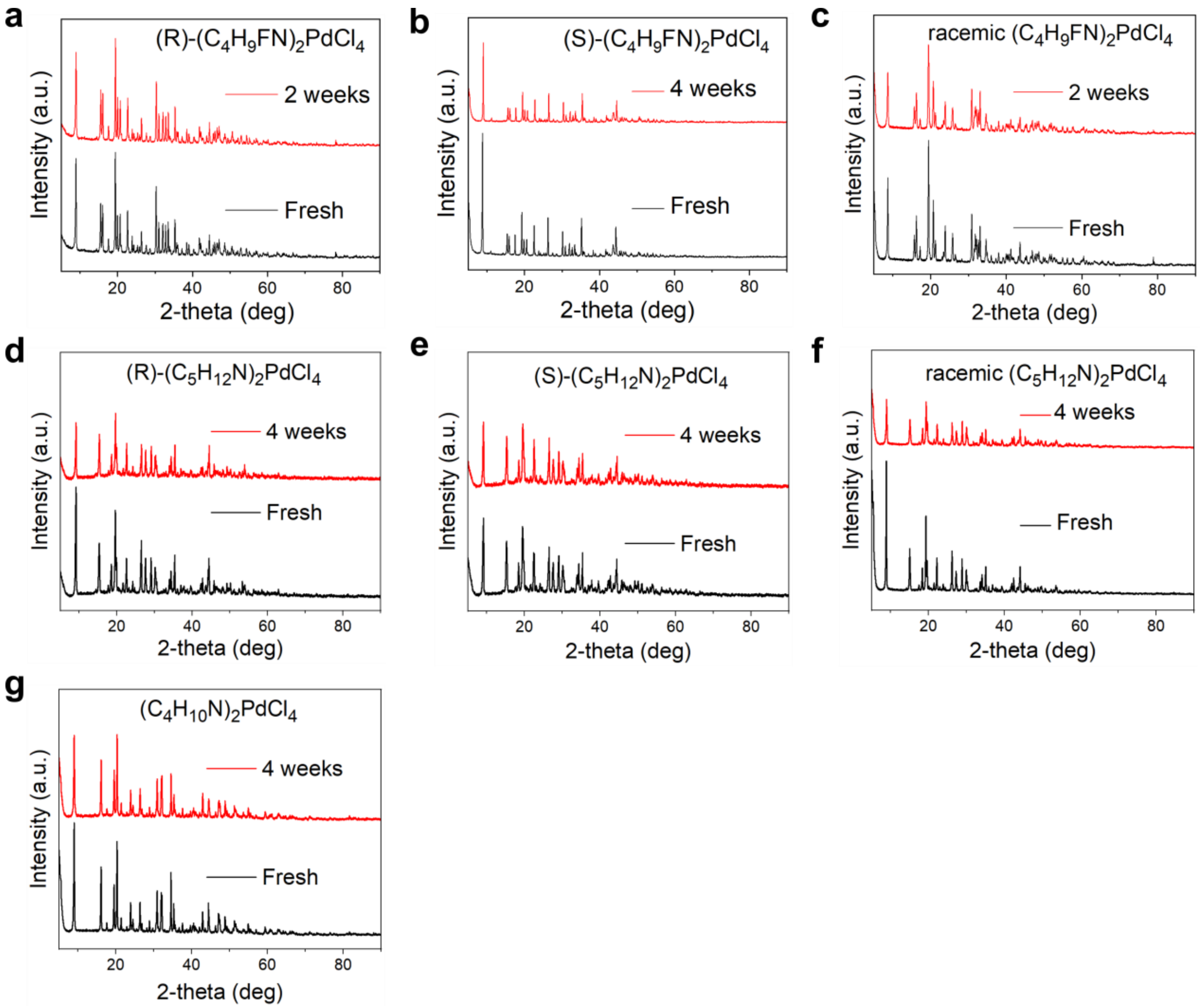

Figure S13. PXRD pattern obtained over time to monitor the air stability of **(a)** R-$(C_4H_9FN)_2PdCl_4$, **(b)** S-$(C_4H_9FN)_2PdCl_4$, **(c)** racemic $(C_4H_9FN)_2PdCl_4$, **(d)** R-$(C_5H_{12}N)_2PdCl_4$, **(e)** S-$(C_5H_{12}N)_2PdCl_4$, **(f)** racemic $(C_5H_{12}N)_2PdCl_4$, and **(g)** achiral $(C_4H_{10}N)_2PdCl_4$.

5. Absorbance Data

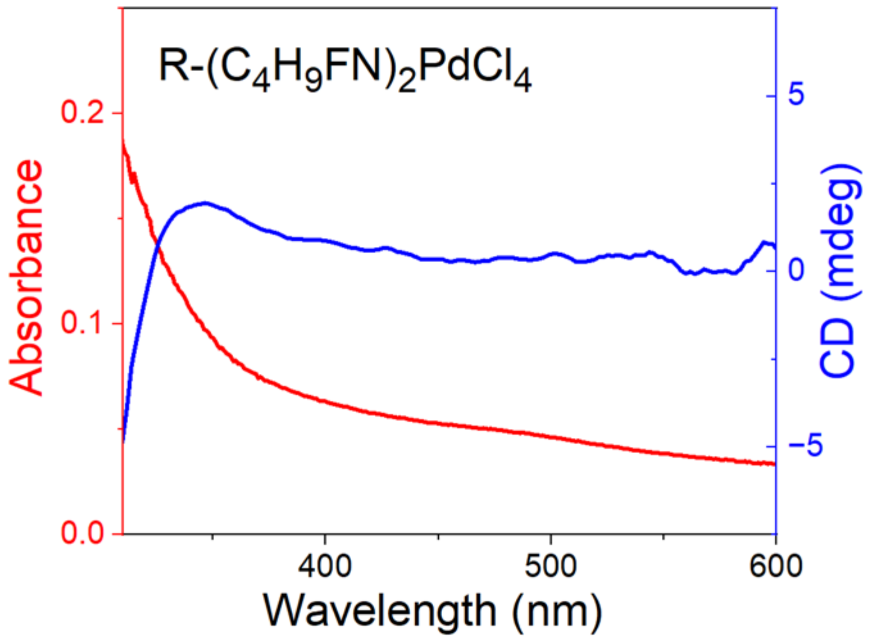

Figure S14. Plot of the obtained UV-Vis absorbance data using R-$(C_4H_9FN)_2PdCl_4$ thin film compared to the CD spectra.
